# Construction of a predictive model for the efficacy of anti-VEGF therapy in macular edema patients based on OCT imaging: a retrospective study

**DOI:** 10.3389/fmed.2025.1505530

**Published:** 2025-03-19

**Authors:** Tingting Song, Boyang Zang, Chui Kong, Xifang Zhang, Huihui Luo, Wenbin Wei, Zheqing Li

**Affiliations:** ^1^Department of Ophthalmology, Shijingshan Teaching Hospital of Capital Medical University, Beijing Shijingshan Hospital, Beijing, China; ^2^School of Clinical Medicine, Tsinghua University, Beijing, China; ^3^School of Information Science and Technology, Fudan University, Shanghai, China; ^4^Beijing Tongren Eye Center, Beijing Ophthalmology and Visual Sciences Key Lab, Beijing Tongren Hospital, Beijing Institute of Ophthalmology, Capital Medical University, Beijing, China; ^5^Foshan Aier Zhuoyue Eye Hospital, Foshan, China

**Keywords:** anti-vascular endothelial growth factor, macular edema, neovascular AMD, retinal vein occlusion, diabetic macular edema, deep learning, ResNet50

## Abstract

**Background:**

Macular edema (ME) is an ophthalmic disease that poses a serious threat to human vision. Anti-vascular endothelial growth factor (anti-VEGF) therapy has become the first-line treatment for ME due to its safety and high efficacy. However, there are still cases of refractory macular edema and non-responding patients. Therefore, it is crucial to develop automated and efficient methods for predicting therapeutic outcomes.

**Methods:**

We have developed a predictive model for the surgical efficacy in ME patients based on deep learning and optical coherence tomography (OCT) imaging, aimed at predicting the treatment outcomes at different time points. This model innovatively introduces group convolution and multiple convolutional kernels to handle multidimensional features based on traditional attention mechanisms for visual recognition tasks, while utilizing spatial pyramid pooling (SPP) to combine and extract the most useful features. Additionally, the model uses ResNet50 as a pre-trained model, integrating multiple knowledge through model fusion.

**Results:**

Our proposed model demonstrated the best performance across various experiments. In the ablation study, the model achieved an F1 score of 0.9937, an MCC of 0.7653, an AUC of 0.9928, and an ACC of 0.9877 in the test conducted on the first day after surgery. In comparison experiments, the ACC of our model was 0.9930 and 0.9915 in the first and the third months post-surgery, respectively, with AUC values of 0.9998 and 0.9996, significantly outperforming other models. In conclusion, our model consistently exhibited superior performance in predicting outcomes at various time points, validating its excellence in processing OCT images and predicting postoperative efficacy.

**Conclusion:**

Through precise prediction of the response to anti-VEGF therapy in ME patients, deep learning technology provides a revolutionary tool for the treatment of ophthalmic diseases, significantly enhancing treatment outcomes and improving patients’ quality of life.

## 1 Introduction

Macular edema (ME) is the leading cause of vision loss in patients (1). The macula is an important central area of the retina that is responsible for ensuring fine vision. Fluid buildup in this area can lead to severe loss of visual function. Retinal vein occlusion (RVO), diabetic retinopathy (DR), and age-related macular degeneration (AMD) are common retinal vascular diseases in developed countries, and they are significant causes of blindness and visual impairment in both working-age and elderly populations. The common pathophysiologic mechanisms that lead to ME in RVO, DR, and AMD include vascular injury, inflammatory response, and disruption of the blood–retinal barrier. Together, these factors lead to increased permeability and fluid accumulation in the retinal vasculature (2–4). This overexpression of growth factors such as VEGF promotes vascular leakage and neovascularization, further exacerbating the pathological process of macular edema (5).

Strategies for the treatment of macular edema (ME) include the use of anti-vascular endothelial growth factor (anti-VEGF) drugs, glucocorticoids, and laser photocoagulation, with the goal of reducing macular edema and improving vision. Among them, anti-VEGF drugs are effective in reducing macular edema and improving vision prognosis by inhibiting the VEGF receptor signaling pathway. Vitreous cavity injection of anti-VEGF drugs has become the first line of treatment for ME, which marks a shift in treatment strategy from merely preserving vision to restoring vision. Despite the revolutionary nature of this treatment, different treatment options and disease severity require frequent injections to be effective. For example, the efficacy of treatment-on-demand (PRN) or treatment-extension (T&E) depends on a subjective judgment of macular central recess edema. This not only places a significant treatment burden on the patient but also carries a risk of complications. At the same time, some patients do not respond completely to these treatments. Effective control of ME or prevention of post-treatment recurrence is essential to preserve vision.

Optical coherence tomography (OCT) is a non-invasive imaging modality for assessing the microstructure of the retinal layers, which is capable of rapidly generating images of ocular tissues and can provide high-resolution cross-sectional images of the retina to help physicians assess the morphologic and structural changes in the macula and to reveal characteristic changes in the retina, such as changes in the thickness of the macula, cyst formation, and subretinal fluid, which can provide an important basis for clinical treatment (6). OCT is widely used to assess treatment efficacy and recurrence in ME, and it is capable of quantifying the thickness of the macular central pucker (7). Although optical coherence tomography (OCT) has a well-established research base for quantitative and qualitative analysis of macular edema (ME), physicians cannot rely solely on these findings to directly predict the long-term severity of the condition and its impact on visual function. At the same time, frequent follow-up visits and examinations increase the burden on patients and strain healthcare resources. Statistics from clinical studies show that patients treated for diabetic macular edema (DME) do not complete a full course of anti-VEGF injections exactly as prescribed (8).

Therefore, exploring the validity of early macular edema morphology in predicting long-term prognosis is necessary for better individualized treatment planning, reducing the number of injections in patients, and predicting the prognosis of patients with varying degrees of macular edema. Manual interpretation of OCT images usually requires a professionally trained ophthalmologist, which involves a certain learning curve and accumulation of experience and is extremely challenging, especially for primary care hospitals. In recent years, artificial intelligence (AI) has shown great potential in the field of medical image analysis; in particular, machine learning (ML) and deep learning (DL) systems have been demonstrated to detect and quantify retinal effusions based on OCT images, which play a standardized quantitative role in the diagnosis and classification of ME (9, 10). This not only accelerates the clinical workflow but also helps to identify patients who require further evaluation or treatment by specialists. Especially in settings with limited healthcare resources, the use of this technology can greatly improve the accessibility and quality of healthcare services. In predicting the recurrence of macular edema associated with retinal vein occlusion (RVO-ME), a multimodal fusion deep migratory learning model using OCT angiography (OCTA) images has been successful. This technique provides new ideas for clinicians to determine the duration of follow-up of patients after anti-VEGF therapy. Nevertheless, the limited size of the database used in the study may affect the model generalization ability and the complex model may lead to overfitting problems (11, 12). The goal of this study was to create a deep learning model that predicts the response to intravitreal anti-VEGF injections in patients with macular edema due to retinal vascular disease. The goal of our study is to develop a deep learning model that is capable of predicting the response to anti-VEGF therapy in patients with ME by analyzing OCT images, thereby providing a more accurate and personalized treatment plan for the clinic. By combining multimodal image data and advanced deep learning techniques, we expect to improve the predictive accuracy of the model and bring innovative improvements to the diagnosis and treatment of ophthalmic diseases.

**Figure 1:**
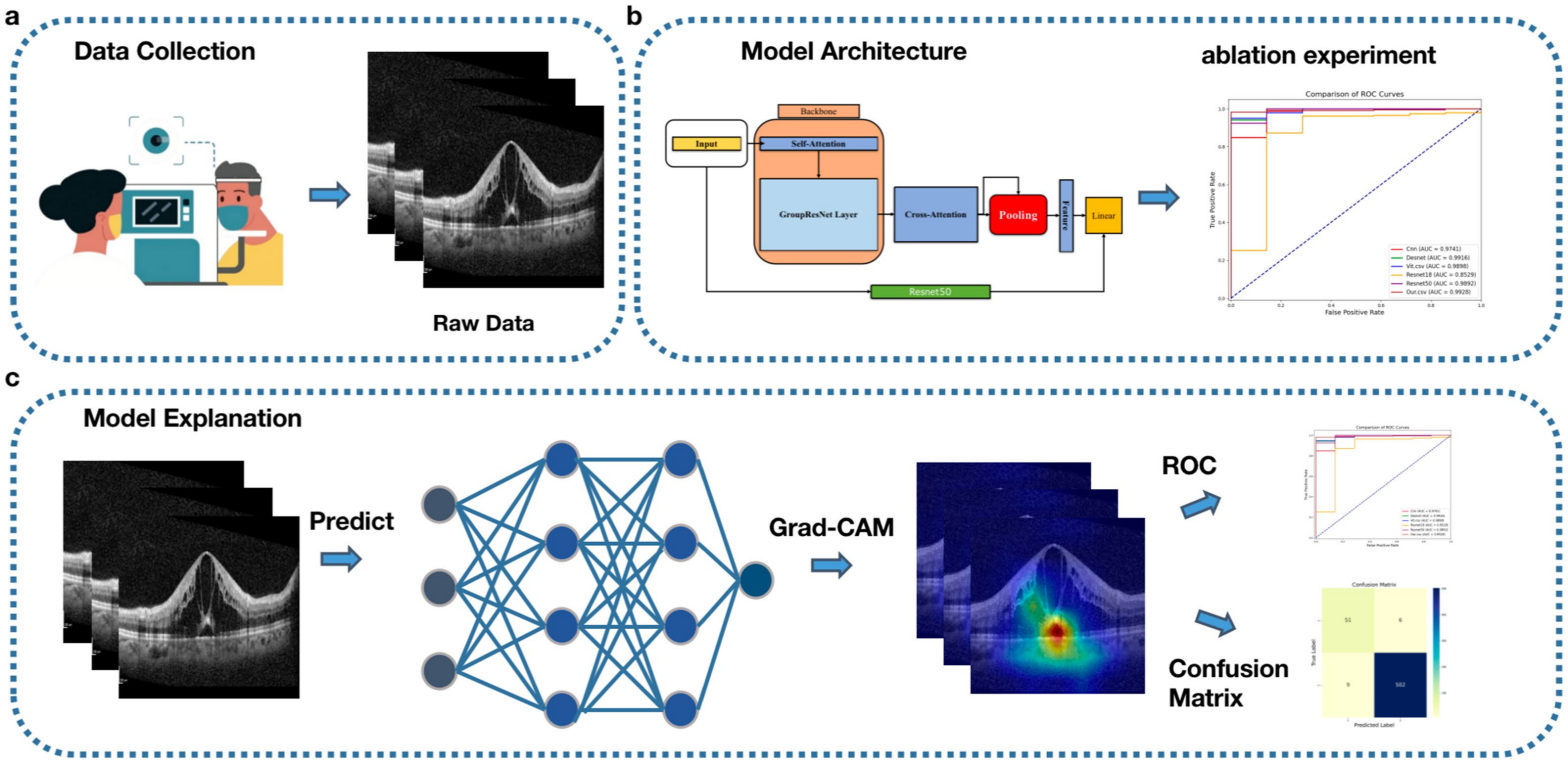
Workflow, **(A)** Data acquisition, collecting patient OCT data. **(B)** Model construction and testing. **(C)** Model visualization.

In this paper, we propose a predictive model for surgical outcomes of macular edema patients based on deep learning and OCT imaging, combining traditional attention mechanisms, grouped convolution, and SPP techniques, and using ResNet50 for model fusion. Experimental results show that the model performs well in the binary categorization task on the first day, first month, and third month after surgery, and the effectiveness of grouped convolution and SPP is verified by ablation experiments. To address the category imbalance problem, a weighting strategy and sampling technique are introduced to significantly enhance the recognition of minority class samples. The model performs well in the diagnosis and grading process and has potential clinical applications that can provide reliable support for the postoperative efficacy assessment of macular edema patients and help optimize personalized treatment plans to improve patient prognosis (Figure 1 shows the workflow).

## 2 Methods

### 2.1 Patient collection

#### 2.1.1 General information

Seventy-two patients (72 eyes) with RVO, DR, and CNV combined with ME admitted to the Department of Ophthalmology of Beijing Shijingshan Hospital from January 2023 to May 2024 were retrospectively collected for the study. The study followed the Declaration of Helsinki, which was reviewed and approved by the ethical committee of Beijing Shijingshan Hospital (Ethical Number: 2024–22), and the enrolled patients signed an informed consent form.

The inclusion criteria for this study were as follows: (1) decreased central visual acuity, with corrected visual acuity ranging from 0.01 to 0.8; (2) presence of macular edema and/or combined choroidal neovascularization, as shown on OCT, including retinal vein occlusion, diabetic retinal disease, and various types of choroidal neovascularization secondary to macular edema with a CST > 250 μm; (3) age between 20 and 91 years; and (4) no history of vitrectomy and high compliance. The exclusion criteria were as follows: (1) those with systemic diseases that could not tolerate anti-VEGF treatment; (2) those with combined ocular trauma, inflammation, and tumor, after vitrectomy treatment; (3) those with other underlying fundus diseases that affect the measurement of the thickness of the macular area, such as macular anterior membranes, macular vitreous pulling syndrome, macular splitting, and macular lentigines; (4) those with silicone oil in the eyes; (5) those with psychiatric disorders; and (6) pregnant women.

#### 2.1.2 Screening methods

Fundus fluorescein angiography combined with fundus indocyanine green angiography (FFA + ICGA) (HRA, Heidelberg Engineering, Germany) or optical coherence tomography angiography (OCTA) (Heidelberg Engineering, Germany) were used to determine the ME criteria.

FFA + ICGA was performed in patients with normal liver and renal function, and after a negative skin test for fluorescein sodium, the pupils of the eyes were dilated with compound tropicamide eye drops. After the pupils were sufficiently dilated, the pupils were examined using Heidelberg fundus fluorescein angiography (Heidelberg Engineering, Germany), and intravenous fluorescein sodium (20%, 3 mL) combined with indocyanine green (2 mL) was injected, and all the examinations were performed by a physician with 12 years of work experience. The judgment criteria were as follows: a. cystic edema, with cystic or even petal-like fluorescent leakage in the macula; b. diffuse edema, with diffuse hyperfluorescent leakage in the posterior pole involving the macula; and c. limited edema, with mottled flaky hyperfluorescent leakage in the macula. The presence of retinal ischemia and choroidal neovascularization leakage was also observed. Patients who could not tolerate fundus fluorescein angiography and had a good refractive interstitial quality were examined using OCTA to determine the presence of neovascularization in the choroidal area, the density of blood flow in each layer of the retina, the presence of non-perfused areas, the presence of microangiomas, and the presence of macular edema. The patients were seated, and a rectangular scan of the posterior pole was performed with the macular central sulcus as the center.

All patients completed the Heidelberg Spectralis OCT (Heidelberg Engineering, Germany) to further define the extent, degree, and cause of ME. Frequency-domain optical coherence tomography (OCT) scanning was performed using a star-shaped scan with fine encryption. Scanning images were superimposed 100 times to obtain the highest thickness within a 1 mm^2^ area of the macular central concavity (measured as the vertical distance from the inner limiting membrane to Bruch’s membrane), which was recorded as the macular central subfield thickness (CST). Each patient was scanned at 48 levels to complete the ME or CNV coverage of the maximum extent of the macular lesion. ME was determined as: a. cystic edema, cystic hypoechoic areas within the neuroepithelium; b. cystic degeneration, elliptical hypoechoic areas of varying sizes within the neuroepithelium; and c. diffuse edema and thickening, with a lack of structural hierarchy of the neuroepithelial layer and overall thickening. After scanning, the CST was measured using the tool provided by the system, with a CST >250 μm used as the criterion for determining ME. The diagnosis of ME was confirmed by two physicians.

#### 2.1.3 Treatment regimen

A treatment regimen was developed for patients who met the enrollment criteria. Anti-VEGF injections are given once a month for three consecutive months. Ranibizumab (RBZ) 0.5 mg/0.05 mL intravitreal intraocular injection (IVI) was administered, with follow-up visits scheduled once a month for 3 months. BCVA and CST were recorded on day 1, month 1, and month 3 after anti-VEGF treatment. The effectiveness of the treatment was assessed by comparing these measurements to the baseline at each of the three time points. The criteria for improvement were as follows: (1) a decrease in CST on OCT compared to the last follow-up; (2) a decrease in intraretinal or subretinal fluid on OCT; and (3) an improvement or no change in naked-eye visual acuity. The criteria for no improvement were: (1) a significant increase in CST on OCT compared to the baseline, or the presence of hemorrhage or ischemia; (2) worsening of the structural disorder in the macular layers on OCT compared to the previous scan; and (3) a decrease in naked-eye visual acuity.

### 2.2 Data preprocessing

In order for the model to adapt and recognize the variable medical images in the real world, we performed data enhancement techniques, such as rotation, translation, and reflection, to the OCT images used for training. These operations were designed to mimic the variations that may be encountered in real OCT image acquisition, allowing the model to recognize the key features even if the images are rotated or shifted. At the same time, we made sure that all images were resized appropriately so that the model could process them uniformly.

### 2.3 An efficacy prediction model based on OCT images and deep learning

#### 2.3.1 Introducing related models

Packet convolution was first used on AlexNet to distribute models across multiple GPUs as an engineering compromise. Later, however, the use of models such as ResNeXt has shown that this module can be used to improve classification accuracy. Specifically, computational efficiency and feature diversity can be improved by processing convolutional operations in groups, but this approach may fall short of capturing global information (13). Spatial pyramid pooling (SPP) is a pooling layer, proposed by He in 2014 (14), that removes the fixed-size constraints of the network, allowing for the pooling of features and the generation of fixed-length outputs, which are then fed into the fully connected layer (or other classifiers). In other words, we can do some information aggregation at a deeper stage of the network hierarchy (between the convolutional and fully connected layers) to avoid cropping or distorting at the beginning, but this approach has a higher computational complexity.

#### 2.3.2 Our proposed model

**Figure 2:**
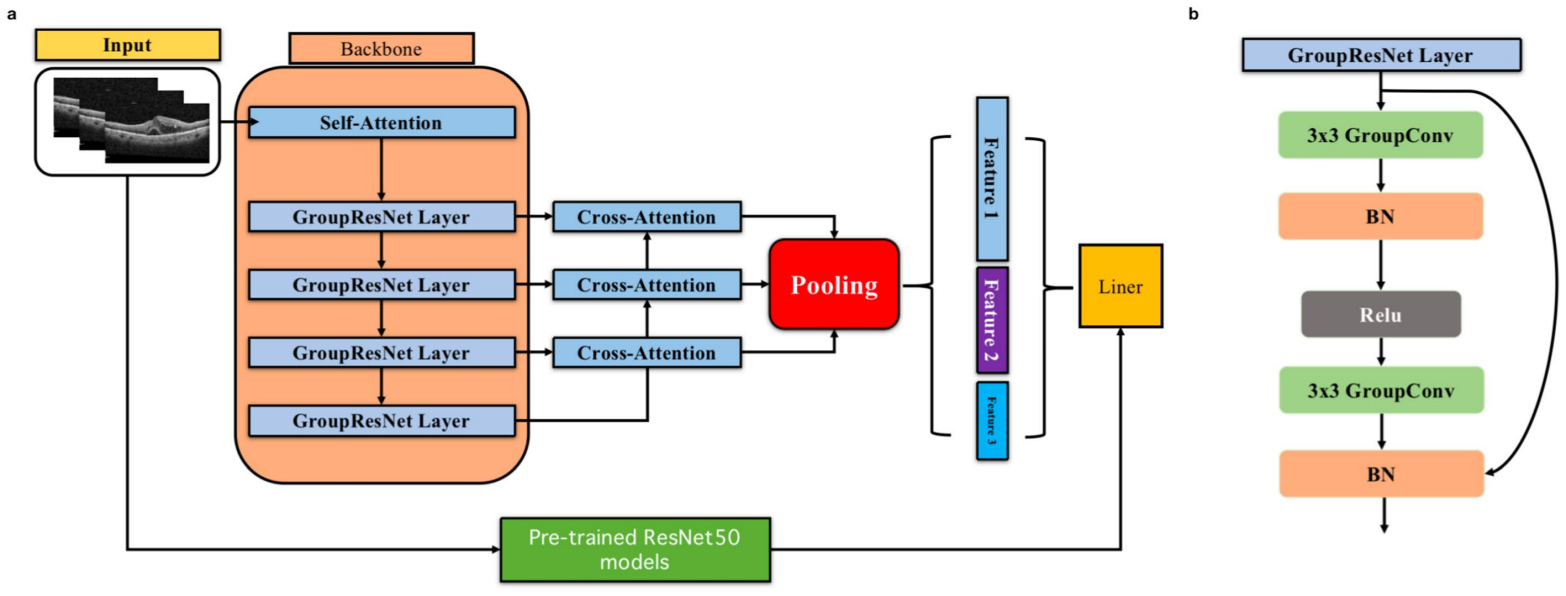
Model design, **(A)** Overall model architecture. **(B)** Group Resnet Layer structure.

To enhance feature extraction and improve prediction accuracy, we propose a deep convolutional neural network that integrates grouped convolution, spatial pyramid pooling (SPP) techniques, and a traditional attention mechanism, with ResNet50 as the pre-trained backbone for model fusion (see Figure 2). This architecture combines the strengths of these techniques while addressing their respective limitations, significantly improving the model’s overall performance and adaptability. Group convolution is a technique for grouping convolution operations, which divides the feature channels into multiple groups and performs convolution operations on each group separately. This method can not only significantly reduce the amount of computation but also increase the diversity of feature extraction. Spatial pyramid pooling is a technique to enhance the ability of multi-scale feature extraction. By performing block pooling operations on the input feature map with different grid sizes, SPP is able to capture information at both local and global levels.

Specifically, ResNet50 is used as the base model to extract global features, and we build upon it by introducing four layers of residual connectivity. These layers include our designed GroupResNetLayer, which incorporates a 3 × 3 grouped convolution, batch normalization (BN), a ReLU activation function, followed by another 3 × 3 grouped convolution, BN, and a residual connection. This structure enhances feature diversity and computational efficiency.

Additionally, we integrate a cross-attention mechanism that allows for dynamic information exchange between the features produced by different GroupResNetLayers. The cross-attention operation emphasizes critical features by assigning weights to the interdependent feature maps, which is particularly important for identifying subtle pathological changes in OCT images. For optimal performance, the feature map resolution is set to 32 × 32, and the number of feature channels is set to 128, balancing computational cost and representation power.

The attention outputs are then merged and subjected to a pooling operation, which leverages SPP to aggregate multi-scale features. Finally, all extracted features are concatenated and input into the final classification layer, ensuring that both local and global information contribute to the predictive outcome. This carefully designed architecture effectively captures and processes complex OCT features, resulting in superior predictive performance, as demonstrated in our experimental results.

#### 2.3.3 Model comparison

In this study, we present a predictive model for surgical outcomes of macular edema patients based on OCT imaging and deep learning and compare it with several currently used models. Existing studies have applied several models to solve problems related to macular edema surgery. For example, generative adversarial networks (GANs) were used to predict the long-term outcome of patients with diabetic macular edema, demonstrating its potential to generate high-quality post-processed OCT images (15). Tang et al. successfully detected and classified macular edema by analyzing the images from multiple commercial OCT devices using a multi-task deep learning system, demonstrating a high degree of DME detection and central DME differentiation with high accuracy; furthermore, the U-Net model improved early recognition after surgery in a macular edema segmentation task (16). Another study used multiple machine learning algorithms to predict visual acuity recovery after anti-VEGF treatment and achieved good predictive performance (17).

In contrast to these models, our model combines grouped convolution and spatial pyramid pooling (SPP) techniques and uses ResNet50 as a pre-trained model for fusion. While retaining the advantages of other models, our model improves the computational efficiency and diversity of feature representations through grouped convolution, SPP enhances the extraction of multi-scale features, and the attention mechanism strengthens the focus on key features. These designs enable our model to have higher potential and reliability in predicting macular edema surgical outcomes. Compared to GANs, our model not only enhances and refines OCT images for better visualization but also provides predictive insights into surgical efficacy. Compared to multi-task deep learning systems, our model excels in multi-scale feature extraction and key feature focus. Compared to U-Net and traditional machine learning algorithms, our model shows improvement in terms of computational efficiency and feature representation, validating its potential value and effectiveness in clinical applications.

### 2.4 Experimental setup

In the process of validating the model’s effectiveness, we conducted three dichotomous experiments at the following time points: the first day after surgery, the first month after surgery, and the third month after surgery, to determine whether the patient had improved (yes/no). These experiments involved comparing our model with other deep learning models while gradually introducing innovations to verify the performance improvement effect of each innovation. We used the ResNet50 pre-trained model as the base model and then gradually introduced grouped convolution and SPP to observe performance changes. Binary classification and ablation experiments at these three time points were used to validate the effectiveness of the proposed model and to demonstrate the important role of grouped convolution and SPP in performance improvement. Prior to training, we partitioned the dataset into training and test sets with a ratio of 8:2. The partitioned dataset was used for training, test comparison experiments, and ablation experiments. The ultimate goal of this study was to comprehensively validate the performance of the model in the efficacy prediction process and to demonstrate that each model component is effective in improving the model performance.

To support the training and evaluation of this model, we performed all tasks in Windows 11 operating system and used a CPU with AMD Ryzen 75800H (16 GB RAM) and a GPU with GeForce RTX™ 4,090 (24 GB RAM) for computation. The experimental programming language used was Python 3.10, and the deep learning framework used was PyTorch version 1.8.0.

### 2.5 Statistical analysis

Evaluation metrics are critical for assessing the performance of machine learning models. These quantitative metrics are essential for objectively assessing the performance of report generation models and guiding their development and improvement. In this experiment, we used a variety of metrics to measure the strengths and weaknesses of each model, including accuracy (ACC), precision (Precision), recall (Recall), F1 value (F1), and area under the subject’s work characteristic curve (AUC), which were obtained by calculating the relationship between true positives (TP), false negatives (FN), true negatives (TN), false positives (FP), AUC, and other metrics.

Accuracy: the percentage of correctly predicted results in the total sample.


$$\mathrm{Accuracy}=\frac{\left(TP+TN\right)}{\left(TP+TN+FP+FN\right)}$$

Precision: the probability of all samples that are predicted to be positive actually being positive.


$$\mathrm{Precision}=\frac{TP}{\left(TP+FP\right)}$$

The Matthews correlation coefficient (MCC) takes into account the four scenarios of true positive (TP), false negative (FN), true negative (TN), and false positive (FP) and is able to provide a balanced assessment of performance.


$$MCC=\frac{TP*TN-FP*FN}{\sqrt{\left(TP+FP\right)\left(TP+FN\right)\left(TN+FP\right)\left(TN+FN\right)}}$$

F1, combining the performance of both precision and recall:


$$F1=\frac{2*\mathrm{Precision}*\mathrm{Recall}}{\left(\mathrm{Precision}+\mathrm{Recall}\right)}$$

In addition to this, we also plot ROC curves and confusion matrices to visualize the model results. The ROC curves are graphs that assess the performance of the classification model by plotting the true positive rate (TPR) and the false positive rate (FPR).

In addition, the data were analyzed using SPSS 22.0 statistical software for the patients’ baseline information. Measurement information that obeyed normal distribution with chi-square was expressed as mean ± standard deviation and paired-sample *t*-test was used for pre- and post-treatment comparisons. Measurement information that did not obey normal distribution was expressed as median and interquartile spacing, and the paired-sample Wilcoxon test was used for comparison before and after treatment. The above differences were considered statistically significant at a *p*-value of ≤0.05.

## 3 Results

### 3.1 Basic patient statistics

#### 3.1.1 Statistical results of patients’ baseline information

The statistical results of baseline information are shown in Table 1. The basic information of the study subjects was as follows: disease distribution was 24 eyes in DME patients, 34 eyes in RVO-ME patients, and 13 eyes in CNV-ME patients; 36 right eyes and 35 left eyes; gender distribution was 40 male patients and 31 female patients; and the age range was from 21 to 91 years old, with a mean age of 59.45 ± 13.27 years. The pre-treatment central retinal thickness (CST) ranged from 252 to 954 μm, with a mean of 568.00 ± 21.46 μm; the standardized visual acuity chart showed that the pre-treatment best-corrected visual acuity (BCVA) Log MAR values ranged from −2.00 to −0.10, with a mean of −0.88 ± 0.05. The intraocular pressure (IOP) was examined using a non-contact tonometer (Full Auto IOP) manufactured by Canon, Japan, and the results showed that the patients’ intraocular pressure was within the normal range. No ocular adverse events such as subconjunctival hemorrhage, endophthalmitis, retinal detachment, or systemic serious adverse events such as cardiovascular and cerebrovascular accidents occurred during the treatment.

**Table 1:** Comparison of BCVA and CST before and after treatment.

After treatment item		*N* (cases)	Pre-treatment	Post-treatment	*Z*-value	*p*-value
24 h	BCVA	50	−0.82(−1.30 ~ −0.60)	−0.60(−1.00 ~ 0.52)	−5.59	<0.001
	CST		601.50(442.25 ~ 716.25)	435.50(336.00 ~ 534.25)		
1 m	BCVA	49	−0.70(−1.30 ~ −0.65)	−0.52(−1.00 ~ −0.40)	−5.37	<0.001
	CST		586.00(428.50 ~ 689.00)	326.00(241.50 ~ 427.00)		
3 m	BCVA	27	−0.70(−1.00 ~ −0.60)	−0.40(−0.70 ~ −0.30)	−4.16	<0.001

Remarks: the difference between BCVA and CST at 24 h and 1 month after treatment and before treatment, and the difference between BCVA and before treatment at 3 months after treatment did not follow a normal distribution. Statistical significance was determined using the Wilcoxon test, a non-parametric test.

#### 3.1.2 Comparison of treatment improvement at three time points after treatment

The number of cases completing the OCT examination on the first day after treatment was 60, with 50 cases showing improvement and 1 case showing no improvement. On the first month after treatment, 54 cases completed the OCT examination, with 49 cases showing improvement and 5 cases showing no improvement. By the third month after treatment, 43 cases completed the OCT examination, with 27 cases showing improvement and 16 cases showing no improvement. In the improved patients, CST decreased and BCVA increased at all three-time points after treatment compared to before treatment, and the differences were statistically significant. Among the degrees of CST decrease, the average decreases at 24 h, 1 month, and 3 months after treatment were 135.78, 220.78, and 238.96 μm, respectively (as shown in Table 2).

**Table 2:** Comparison of BCVA and CST before and after treatment.

After treatment Item		*N* (cases)	Pre-treatment	Post-treatment	t-value	*p-value*
3 m	CST	27	572.81 ± 182.11	333.85 ± 147.14	−5.86	<0.001

The difference between CST at 3 months after treatment and before treatment follows a normal distribution, and the difference is statistically significant using the paired-sample *t*-test.

### 3.2 Results of model ablation experiments

**Figure 3:**
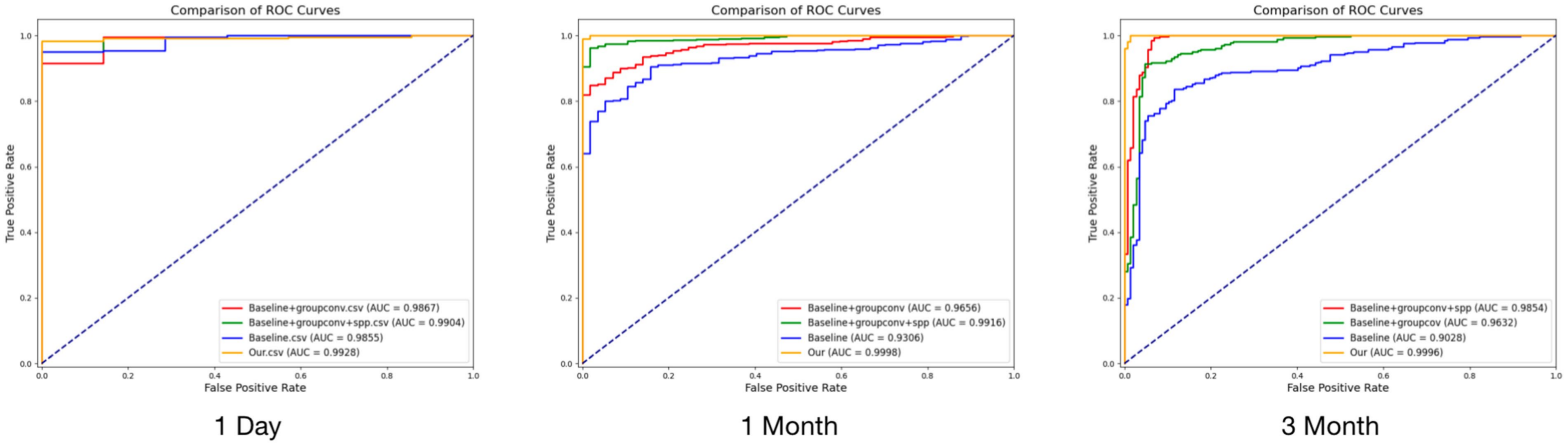
Results of ablation experiments at three time points.

The results of the ablation experiments showed that the gradual introduction of grouped convolution and SPP techniques significantly improved the model performance (see Figure 3 and Table 3). In the test on the first postoperative day, compared to the baseline model, the F1, MCC, AUC, and ACC were 0.7559, 0.2062, 0.9855, and 0.6189, respectively, and the metrics were somewhat improved by adding grouped convolution, and the performance was further improved by combining SPP. Eventually, our model reached the highest values across all metrics, with F1 at 0.9937, MCC at 0.7653, AUC at 0.9928, and ACC at 0.9877. Similar enhancements were verified in the tests in the first and third postoperative months, and the final model exceeded the baseline model in all metrics. Overall, the introduction of grouped convolution and SPP techniques substantially improved the predictive performance of the model, validating the effectiveness of these techniques in processing OCT images and predicting the efficacy of macular edema after surgery.

**Table 3:** Results of ablation experiments for the proposed model.

Test	Model	F1	MCC	AUC	ACC
First day	Baseline	0.7559	0.2062	0.9855	0.6189
	Groupconv	0.9697	0.4998	0.9867	0.9426
	Groupconv + SPP	0.9851	0.6421	0.9904	0.9713
	Our	0.9937	0.7653	0.9928	0.9877
First month	Baseline	0.9422	0.5847	0.9306	0.8996
	Groupconv	0.9427	0.6262	0.9656	0.9014
	Groupconv + SPP	0.9853	0.8574	0.9916	0.9736
	Our	0.9961	0.9980	0.9998	0.9930
Third month	Baseline	0.8820	0.6780	0.9028	0.8471
	Groupconv	0.9180	0.7812	0.9632	0.8938
	Groupconv + SPP	0.9786	0.9304	0.9854	0.9703
	Our	0.9939	0.9803	0.9996	0.9915

Group Convolution partitions input channels into separate groups, each processed independently, reducing computational complexity and enhancing feature independence. Spatial Pyramid Pooling (SPP) employs multi-scale pooling and feature concatenation, enabling the network to handle varying input sizes and improve multi-scale feature extraction.

### 3.3 Model comparison experimental results

**Figure 4:**
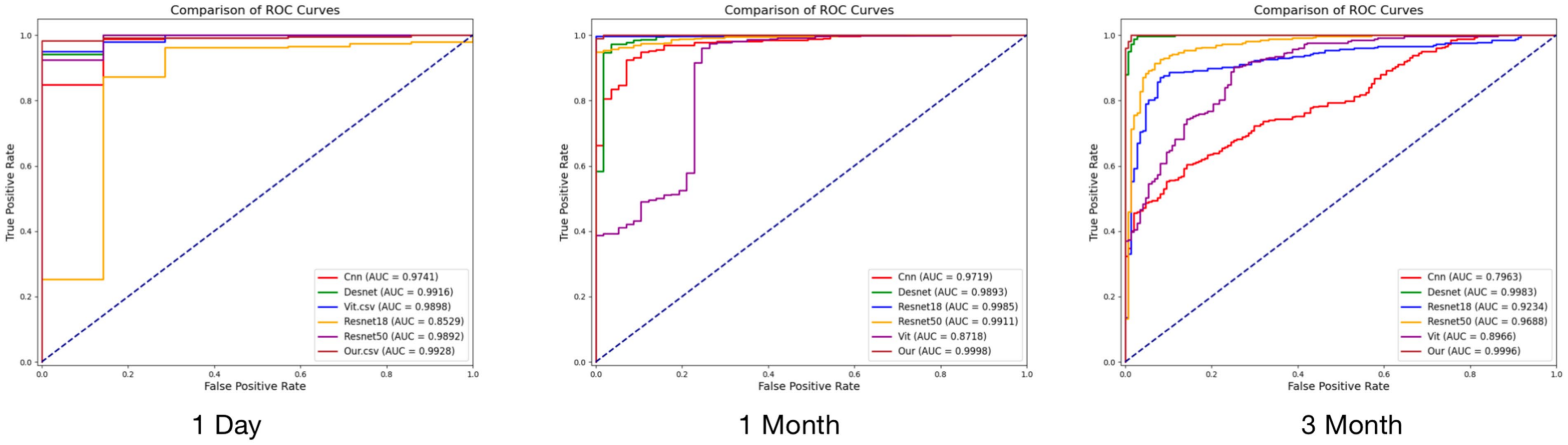
Comparative experimental results at three time points.

Our proposed new model performed well in tests at different postoperative time points (see Figure 4 and Table 4 for details). On the first postoperative day, the model’s F1, MCC, AUC, and ACC reached 0.9937, 0.7653, 0.9928, and 0.9877, respectively. On the first postoperative month, the F1 and MCC were 0.9961 and 0.998, respectively, and in the test on the third postoperative month, the model’s F1 and AUC were 0.9939 and 0.9996. These results indicate that our model achieved excellent performance in prediction at all time points, showing excellent accuracy and reliability.

**Table 4:** Comparison of the performance of the models.

Test	Model	F1	MCC	AUC	ACC
First day	ResNet18	0.9854	0.9713	0.8529	0.9713
	VIT	0.9854	0.9713	0.9898	0.9713
	CNN	0.8000	0.2329	0.9741	0.6762
	Desnet	0.9348	0.4131	0.9916	0.8811
	Resnet50	0.9652	0.4725	0.9892	0.9344
	Our	0.9937	0.7653	0.9928	0.9877
First month	ResNet18	0.9672	0.7237	0.9719	0.9419
	VIT	0.9951	0.9541	0.9985	0.9912
	CNN	0.8633	0.3701	0.8718	0.7782
	Desnet	0.955	0.7182	0.9911	0.9225
	ResNet50	0.9832	0.848	0.9893	0.9701
	Our	0.9961	0.998	0.9998	0.993
Third month	ResNet18	0.6449	0.4202	0.7963	0.6306
	VIT	0.9147	0.7566	0.9234	0.8875
	CNN	0.8706	0.5141	0.8966	0.7962
	Desnet	0.9406	0.8155	0.9688	0.9193
	ResNet50	0.986	0.9558	0.9983	0.9809
	Our	0.9939	0.9803	0.9996	0.9915

In this study, the version details of CNN, ViT, and DesNet are LeNet-5, ViT-B/16 (pre-trained), and DenseNet-121, respectively.

Comparing the results of the other models, on the first postoperative day, the F1 and ACC scores for ResNet18 and ViT were approximately 0.9854 and 0.9713, while DenseNet and ResNet50 performed slightly worse. In the first postoperative month, the F1 scores for ViT and ResNet50 were 0.9951 and 0.9832, respectively, with DenseNet and ResNet18 performing slightly worse. Three months after surgery, the F1 score and AUC value for ResNet50 were 0.986 and 0.9983, respectively, followed by ViT and DenseNet. ResNet18 and CNN performed relatively poorly. In contrast, our proposed model shows superior performance at all time points and on most metrics, validating its superior performance in processing OCT images and predicting postoperative outcomes.

### 3.4 Model interpretability analysis

**Figure 5:**
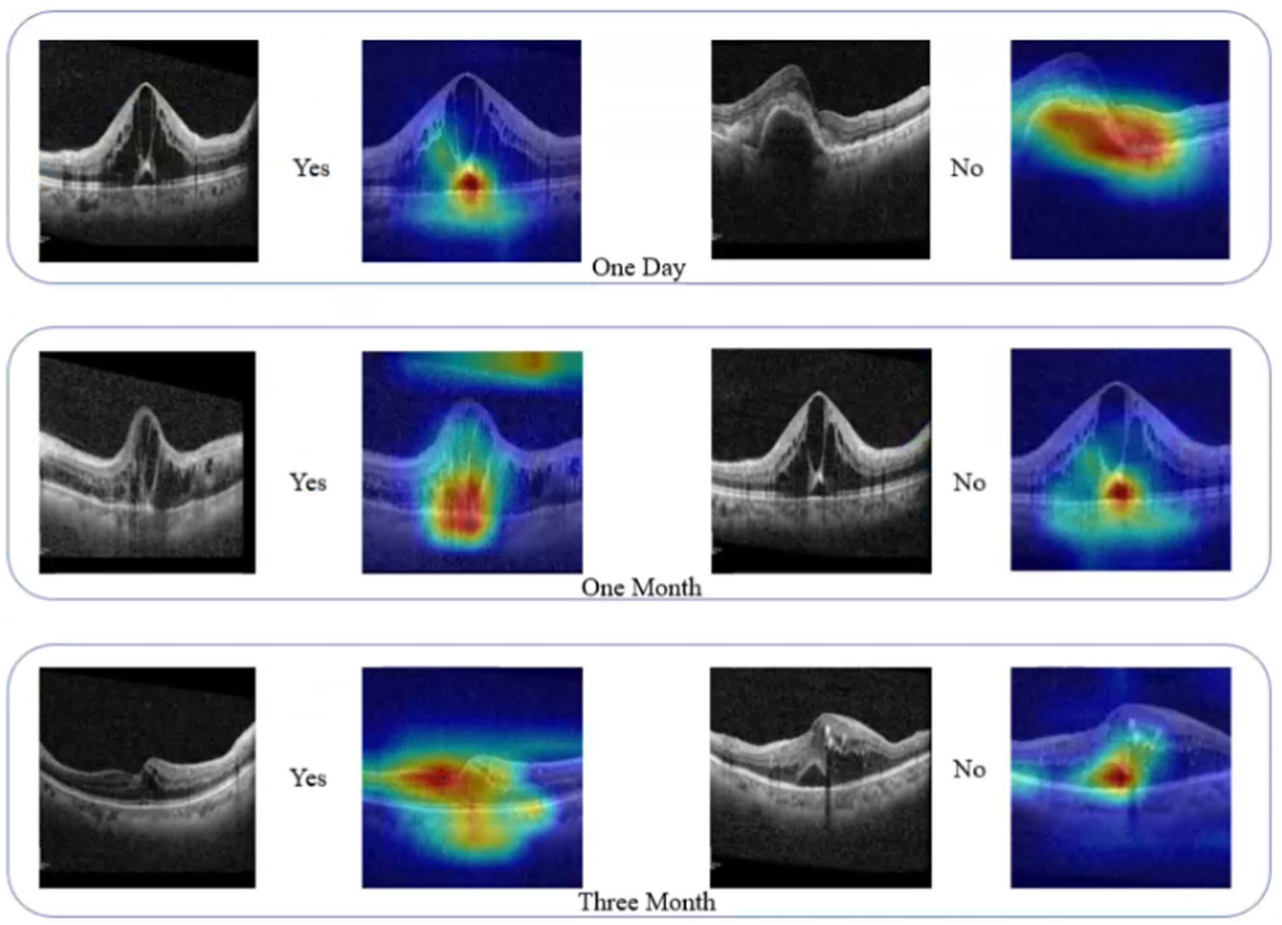
Activation heatmap visualization region for deep learning models.

The model we constructed can be used to interpret the prediction results using Grad-CAM visualization technology, highlighting the image regions that the model focuses on, thus helping us to understand the decision-making process of the model. In the prediction task of OCT images, Grad-CAM was able to clearly show the lesion regions involved in the model’s prediction (see Figure 5), which not only validated the accuracy of the model but also provided valuable clinical insights.

## 4 Discussion

In this study, we proposed a deep convolutional neural network model that integrates group convolution and spatial pyramid pooling (SPP) for predicting postoperative efficacy in patients with macular edema. In ablation experiments, our model achieved F1, MCC, AUC, and ACC scores of 0.9937, 0.7653, 0.9928, and 0.9877, respectively, on the first postoperative day, significantly outperforming other models. In comparison experiments, the model achieved ACC scores of 0.9930 and 0.9915 in the first and third months after surgery, with AUC values of 0.9998 and 0.9996. These experimental results demonstrate that our model exhibited excellent predictive performance at various time points, confirming its superiority in processing OCT images and predicting postoperative efficacy, and highlighting its significant clinical application value (15–17).

The superior performance of our model is primarily attributed to the high compatibility between the model architecture and the prediction task. The model combines traditional attention mechanisms, group convolution, and spatial pyramid pooling (SPP) techniques, using ResNet50 for pre-training and fusion. Group convolution improves computational efficiency and feature diversity, while SPP enhances multi-scale feature extraction capabilities. Weighted strategies and sampling techniques effectively addressed the issue of class imbalance. When processing OCT images, the model accurately captures subtle pathological changes in the images through multi-scale feature extraction and attention mechanisms, which is especially important for detecting subtle changes in macular edema. Specifically, the group convolution in our model improves computational efficiency and enhances feature diversity; the SPP technology significantly improves the ability to capture lesion features through multi-scale feature pooling; and the cross-attention mechanism further strengthens the information interaction between different features. The synergy of these modules enables our model to perform well in microstructural analysis and lesion localization of OCT images. In particular, by combining traditional architectures such as ResNet50, our model achieves a good balance between complexity and performance.

The clinical interpretability of the model was significantly enhanced through Grad-CAM technology. The heatmaps generated by Grad-CAM revealed the regions of the OCT images the model focused on when making predictions, providing insights into the model’s decision-making basis. This visualization method allows clinicians to intuitively observe the key pathological areas the model focuses on, such as the specific location of macular edema, enhancing trust in the model’s predictions. The heatmaps generated by Grad-CAM were consistent with clinical observations, validating the model’s effectiveness. By clearly identifying the regions of interest, clinicians can better understand and verify the model’s predictions, improving the accuracy and efficiency of clinical decision-making.

RBZ is a recombinant monoclonal antibody fragment that has been shown to effectively inhibit vascular endothelial growth factor (VEGF) (18). Previous studies have mainly analyzed baseline factors before treatment to predict the short-term or long-term efficacy of anti-VEGF therapy in ME (19, 20). A pre-treatment study (21) indicated that ranibizumab IVI significantly reduced VEGF levels in the aqueous humor within 24 to 72 h post-injection. Therefore, this study uses OCT imaging at 24 h post-treatment to assess the long-term efficacy of therapy, including the extent and severity of macular edema, the area and morphology of CNV, and other factors. In this study, we established clear criteria for evaluating treatment outcomes. Improvement was defined as a reduction in central retinal thickness (CST) compared to the previous follow-up, as observed by optical coherence tomography (OCT), with a reduction in intraretinal or subretinal fluid and an improvement or stabilization of visual acuity. Conversely, if OCT showed a significant increase in CST from baseline, accompanied by hemorrhage or ischemia, exacerbation of structural disorganization in the macula, or a decline in visual acuity, the treatment was considered ineffective. These criteria helped us objectively evaluate the efficacy of the treatment.

This study observed the recovery level of patients 3 months after anti-VEGF treatment using a monthly intravitreal injection (IVI) regimen, which was consistent with previous classical studies (22). The study showed that after anti-VEGF treatment, the central retinal thickness (CST) decreased significantly at 3 weeks, which is consistent with the 1-month observation in this study. A review study reported that for every 100 μm decrease in CST, the logMAR visual acuity increased by 0.21. In this study, the 24-h CST decreased by 166 μm, and the logMAR visual acuity increased by 0.22, which was basically consistent with Ref. (2). This shows that anti-VEGF treatment has a rapid and positive effect on ME patients and can improve retinal conditions in a short period of time. This treatment effect is consistent with the results of previous studies and further confirms the effectiveness of anti-VEGF therapy in the treatment of ME. Although anti-VEGF treatment significantly improves ME and enhances visual function in patients, persistent macular edema, refractory macular edema, and non-responsive cases prevent patients from achieving a good prognosis even after multiple consecutive IVIs (2). At the same time, anti-VEGF can only inhibit the development of neovascular glaucoma (NVG) in RVO-ME but cannot prevent it (23). The core goal of this study is to explore the relief of macular edema within 24 h after anti-VEGF therapy, using this as a key indicator to predict the potential response value of patients to long-term treatment through model analysis. For patients with poor prognosis identified through early screening, this study advocates the early adoption of combined treatment plans to enhance the treatment effect, improve the patient’s visual prognosis, and correspondingly reduce the economic burden caused by the disease. Through this study, we hope to provide clinicians with more accurate treatment decision support and accurately grasp the individualized treatment needs of patients.

This study focused on analyzing the therapeutic effects of treating macular edema (ME) caused by retinal vascular diseases; however, it has some limitations. First, the sample size is restricted by logistical challenges in completing OCT examinations within 24-h post-surgery and by the single-center data source, limiting generalizability across different imaging protocols and devices. Second, differences in pathophysiological processes among the three included disease types (DME, RVO, and AMD) may affect model applicability. Future studies should expand the sample size and stratify analyses by disease type. Additionally, incorporating multimodal biomarkers such as systemic or genetic data could enhance the model’s predictive power and sensitivity. Finally, external validation using independent datasets and prospective clinical trials is needed to ensure the model’s robustness and real-world clinical utility.

## 5 Conclusion

This study focused on the short-term efficacy of anti-VEGF therapy in macular edema patients by evaluating changes in central retinal thickness (CST) and best-corrected visual acuity (BCVA) within 24 h before and after treatment. We proposed an innovative deep learning model that combines group convolution and spatial pyramid pooling (SPP) techniques to accurately predict patients’ treatment responses using OCT images. Ablation experiments demonstrated that the model’s performance significantly improved as key technologies were progressively introduced. The results showed that the model exhibited excellent predictive performance in tests conducted on the first day, first month, and third month post-surgery, significantly outperforming traditional methods. Despite these positive results, the study had a small sample size. Future research should expand the sample size and incorporate biomarkers to further optimize the model’s prognostic prediction capabilities. Through this research, we aim to provide more accurate decision support for clinical treatment, ultimately improving patient outcomes and long-term visual prognosis.

## Data Availability

The original contributions presented in the study are included in the article/supplementary material, further inquiries can be directed to the corresponding author.

## References

[1] Petrovic N Todorovic D Sarenac Vulovic T Sreckovic S Zivic F Risimic D. Combined treatment of persistent diabetic macular edema with Aflibercept and triamcinolone Acetonide in Pseudophakic eyes. Medicina (Kaunas). (2023) 59:982. doi: 10.3390/medicina59050982, PMID: 37241214 PMC10220700

[2] Sorour OA Levine ES Baumal CR Elnahry AG Braun P Girgis J. Persistent diabetic macular edema: definition, incidence, biomarkers, and treatment methods. Surv Ophthalmol. (2023) 68:147–74. doi: 10.1016/j.survophthal.2022.11.008, PMID: 36436614

[3] Liberski S Wichrowska M Kocięcki J. Aflibercept versus Faricimab in the treatment of Neovascular age-related macular degeneration and diabetic macular edema: a review. Int J Mol Sci. (2022) 23:9424. doi: 10.3390/ijms23169424, PMID: 36012690 PMC9409486

[4] Tadayoni R Paris LP Danzig CJ Abreu F Khanani AM Brittain C. Efficacy and safety of Faricimab for macular edema due to retinal vein occlusion: 24-week results from the BALATON and COMINO trials. Ophthalmology. (2024) 131:950–60. doi: 10.1016/j.ophtha.2024.01.029, PMID: 38280653

[5] Hang A Feldman S Amin AP Ochoa JAR Park SS. Intravitreal anti-vascular endothelial growth factor therapies for retinal disorders. Pharmaceuticals (Basel). (2023) 16:1140. doi: 10.3390/ph16081140, PMID: 37631054 PMC10458692

[6] Zeppieri M Marsili S Enaholo ES Shuaibu AO Uwagboe N Salati C. Optical coherence tomography (OCT): a brief look at the uses and technological evolution of ophthalmology. Medicina (Kaunas). (2023) 59:2114. doi: 10.3390/medicina59122114, PMID: 38138217 PMC10744394

[7] Szeto SK Lai TY Vujosevic S Sun JK Sadda SR Tan G. Optical coherence tomography in the management of diabetic macular oedema. Prog Retin Eye Res. (2024) 98:101220. doi: 10.1016/j.preteyeres.2023.101220, PMID: 37944588

[8] Ciulla TA Pollack JS Williams DF. Visual acuity outcomes and anti-VEGF therapy intensity in diabetic macular oedema: a real-world analysis of 28 658 patient eyes. Br J Ophthalmol. (2021) 105:216–21. doi: 10.1136/bjophthalmol-2020-315933, PMID: 32265201 PMC7848066

[9] Shahriari MH Sabbaghi H Asadi F Hosseini A Khorrami Z. Artificial intelligence in screening, diagnosis, and classification of diabetic macular edema: a systematic review. Surv Ophthalmol. (2023) 68:42–53. doi: 10.1016/j.survophthal.2022.08.004, PMID: 35970233

[10] Alryalat SA Al-Antary M Arafa Y Azad B Boldyreff C Ghnaimat T. Deep learning prediction of response to anti-VEGF among diabetic macular edema patients: treatment response analyzer system (TRAS). Diagnostics (Basel). (2022) 12:312. doi: 10.3390/diagnostics12020312, PMID: 35204404 PMC8870773

[11] Xu F Yu X Gao Y Ning X Huang Z Wei M. Predicting OCT images of short-term response to anti-VEGF treatment for retinal vein occlusion using generative adversarial network. Front Bioeng Biotechnol. (2022) 10:914964. doi: 10.3389/fbioe.2022.914964, PMID: 36312556 PMC9596772

[12] Li D Ran AR Cheung CY Prince JL. Deep learning in optical coherence tomography: where are the gaps? Clin Experiment Ophthalmol. (2023) 51:853–63. doi: 10.1111/ceo.14258, PMID: 37245525 PMC10825778

[13] Qiao W Xu Y Li H. Lie group convolution neural networks with scale-rotation equivariance. Neural Netw. (2024) 183:106980. doi: 10.1016/j.neunet.2024.106980, PMID: 39626532

[14] He K Zhang X Ren S Sun J. Spatial pyramid pooling in deep convolutional networks for visual recognition. IEEE Trans Pattern Anal Mach Intell. (2015) 37:1904–16. doi: 10.1109/TPAMI.2015.2389824, PMID: 26353135

[15] Baek J He Y Emamverdi M Mahmoudi A Nittala MG Corradetti G. Prediction of long-term treatment outcomes for diabetic macular edema using a generative adversarial network. Transl Vis Sci Technol. (2024) 13:4. doi: 10.1167/tvst.13.7.4 PMC11223618 38958946

[16] Yao J Lim J Lim GYS Ong JCL Ke Y Tan TF. Novel artificial intelligence algorithms for diabetic retinopathy and diabetic macular edema. Eye Vision. (2024) 11:23. doi: 10.1186/s40662-024-00389-y, PMID: 38880890 PMC11181581

[17] Kim EJ Lin WV Rodriguez SM Chen A Loya A Weng CY. Treatment of diabetic macular edema. Curr Diab Rep. (2019) 19:1–10. doi: 10.1007/s11892-019-1188-4, PMID: 31359157

[18] Wykoff CC Brown DM Croft DE Major JC Jr Wong TP. Progressive retinal nonperfusion in ischemic central retinal vein occlusion. Retina. (2015) 35:43–7. doi: 10.1097/IAE.0000000000000277, PMID: 25102193

[19] Yin H Zhong S. Efficacy of ranibizumab combined with photocoagulation for diabetic retinopathy: a meta-analysis study. Medicine (Baltimore). (2023) 102:e34170. doi: 10.1097/MD.0000000000034170, PMID: 37543834 PMC10403042

[20] Lowater SJ Grauslund J Subhi Y Vergmann AS. Clinical trials and future outlooks of the port delivery system with Ranibizumab: a narrative review. Ophthalmol Ther. (2024) 13:51–69. doi: 10.1007/s40123-023-00843-5, PMID: 38055121 PMC10776525

[21] Li S Yang Y Zou J Zeng J Ding C. The efficacy and safety of intravitreal injection of Ranibizumab as pre-treatment for vitrectomy in proliferative diabetic retinopathy with vitreous hemorrhage. BMC Ophthalmol. (2022) 22:63. doi: 10.1186/s12886-022-02303-3, PMID: 35139812 PMC8830025

[22] Uludag G Hassan M Matsumiya W Pham BH Chea S Trong Tuong Than N. Efficacy and safety of intravitreal anti-VEGF therapy in diabetic retinopathy: what we have learned and what should we learn further? Expert Opin Biol Ther. (2022) 22:1275–91. doi: 10.1080/14712598.2022.2100694, PMID: 35818801 PMC10863998

[23] Qu S Zou Y Yang L Wu H. The progress of assessment methods and treatments of neovascular glaucoma secondary to central retinal vein occlusion. Front Med (Lausanne). (2024) 10:1280776. doi: 10.3389/fmed.2023.1280776, PMID: 38259837 PMC10800625

